# Cartography of Free-Living Amoebae in Soil in Guadeloupe (French West Indies) Using DNA Metabarcoding

**DOI:** 10.3390/pathogens9060440

**Published:** 2020-06-04

**Authors:** Yann Reynaud, Célia Ducat, Antoine Talarmin, Isabel Marcelino

**Affiliations:** TReD-Path Unit (Transmission, Réservoirs et Diversité des Pathogènes), LEMic (Laboratoire Interactions des Ecosystèmes Microbiens), Institut Pasteur de la Guadeloupe, Morne Jolivière, 97183 Abymes, France; yreynaud@pasteur-guadeloupe.fr (Y.R.); cducat64@gmail.com (C.D.); ATALARMIN@pasteur-guadeloupe.fr (A.T.)

**Keywords:** soil, free-living amoebae, metabarcoding, Guadeloupe islands

## Abstract

Free-living amoebae (FLA) are ubiquitous protists. Pathogenic FLA such as *N. fowleri* can be found in hot springs in Guadeloupe, soil being the origin of this contamination. Herein, we analyzed the diversity and distribution of FLA in soil using a targeted metataxonomic analysis. Soil samples (n = 107) were collected from 40 sites. DNA was extracted directly from soil samples or from FLA cultivated at different temperatures (30, 37 and 44 °C). Metabarcoding studies were then conducted through FLA 18SrDNA amplicons sequencing; amplicon sequence variants (ASV) were extracted from each sample and taxonomy assigned against SILVA database using QIIME2 and SHAMAN pipelines. *Vermamoeba* were detected in DNA extracted directly from the soil, but to detect other FLA an amoebal enrichment step was necessary. *V. vermiformis* was by far the most represented species of FLA, being detected throughout the islands. Although *Naegleria* were mainly found in Basse-Terre region, *N. fowleri* was also detected in Grand Terre and Les Saintes Islands. *Acanthamoeba* were mainly found in areas where temperature is approx. 30 °C. *Vannella* and *Vahlkampfia* were randomly found in Guadeloupe islands. FLA detected in Guadeloupe include both pathogenic genera and genera that can putatively harbor microbial pathogens, therefore posing a potential threat to human health.

## 1. Introduction

Protists are, by far, one of the most diverse and abundant eukaryotes in soil. Nevertheless, they are the least studied group of soil organisms [1].

Free-living amoebae (FLA) are ubiquitous protists [2]. They can be found in water (natural or man-made) environments such as: wastewater treatment plants [3], drinking water networks [4], tap water [5,6,7], hospital water networks [8,9], hot springs [10,11], swimming pools [12,13], and rivers [14]. Although FLA appear to be omnipresent in soils [15,16], few studies are currently available. So far, FLA have been detected in deserts [17], wet soils [18,19], in sediments [20,21] and in soil from agricultural and mining sites [22]. 

Amongst the most common genera of FLA, some can be pathogenic causing encephalitis (*Acanthamoeba* spp., *Balamuthia mandrillaris*, *Sappinia pedata* and *Naegleria fowleri*) [23]. Although the incidence of human infections by these amoebae is generally low, new cases are being constantly reported worldwide [23,24] *Acanthamoeba* spp. can also cause keratitis. The genera *Acanthamoeba* and *Vermamoeba* also have medical importance as hosts, vehicles, and training grounds for bacteria [25,26].

The diversity and ecology of FLA have been assessed using culture-dependent and independent approaches [2,26]. Cultivation-based approaches are indispensable to estimate the abundance and diversity of the numerically dominant FLA. Nevertheless, morphological identification of amoebae is time-consuming and correct amoeba identification requires specialized expertise. Due to significant differences in cultivability of the diverse FLA (feeding source, temperature, possibility of non-cultivable FLA), it is widely accepted that studies using cultivation approaches alone are likely to underestimate FLA diversity. The use of culture-independent approaches such as PCR or high-throughput sequencing (HTS) has been recently implemented, herewith providing valuable information on the actual diversity of FLA. At the moment, there are no universal primers for the detection of FLA, but various primer pairs have been designed to identify and/or quantify FLAs (including non-cultivable ones) in different environments (reviewed by Samba-Louaka et al., 2019) [2]. Recent advances in HTS and computational biology now allow the exploration of microbial communities based on culture-independent approaches using metagenomics. This enables us to quantify and functionally characterize environmental microbiomes with unprecedented precision and comprehensiveness [27].

The work presented herein aimed at characterizing for the first time the biodiversity and the distribution of FLA in soil in the Guadeloupe islands, using metataxonomic analyses. For this, we collected several wet soil samples, near water sources in Guadeloupe islands, and used novel primers targeting the 18S rRNA gene of FLA. Then, FLA diversities were identified by 18S Illumina sequencing, followed by a high-throughput DNA metabarcoding analysis. To assess FLA diversity, we also tested culture-dependent and independent approaches. 

## 2. Results

### 2.1. Identification of FLA and Relative Aabundance Using Culture-Dependent vs. Independent Approaches

**Figure 1:**
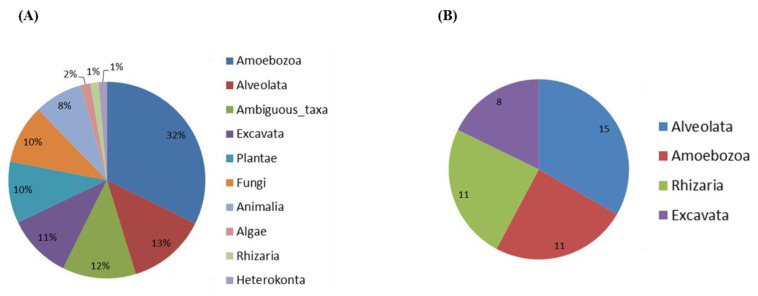
Eukaryote community composition based on high-throughput sequencing of 18S rRNA gene. (**A**) Distribution of overall eukaryotic taxonomic groups. (**B**) Number of genus within protozoan taxa including the supergroups Alveolata, Amoebozoa, Excavata and Rhizaria.

Soil samples were directly used for DNA extraction or used for an amoebal enrichment step at 30, 37 and 44 °C, allowing us to successfully amplify and detect cultivable FLA. Among the 817 Amplicon Sequence Variants (ASV) identified, 59.2% were assigned at family level and 49.2% at genus level. The distribution of eukaryotic sequences generated by Illumina high-throughput sequencing is shown in Figure 1. Proportion of relative abundance varied between 1.3% (minimum) and 32.3% (maximum) of these sequences, and belonged to clades of eukaryotes from the lineages Amoebozoa (32.3%), Alveolata (13%), Excavata (10.6%), Plantae (10.1%), Fungi (9.7%), Animalia (7.9%), Algae (1.6%), Rhizaria (1.4%) and Heterokonta (1.3%); 12.1% were considered as ambiguous taxa (Figure 1A). Within the protozoan taxa, we found amoeba from the supergroups Alveolata, Amoebozoa, Excavata and Rhizaria (Figure 1B). A total of 45 genera were identified within these supergroups. The most abundant genera of FLA were *Vermamoeba* (Amoebozoa), *Naegleria* and *Vahlkampfia* (both Excavata), *Vannella* and *Acanthamoeba* (both Amoebozoa).

**Figure 2:**
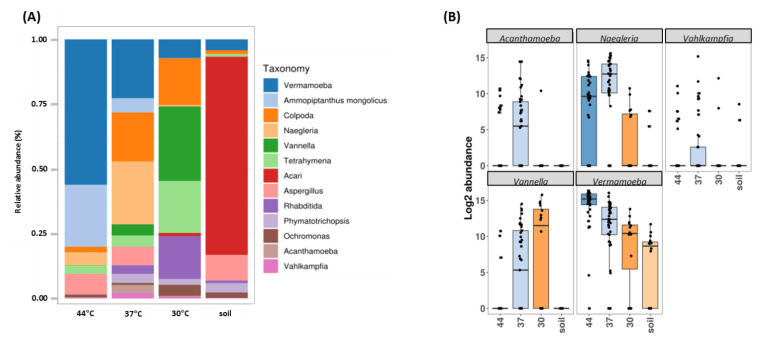
Eukaryote community composition based on high-throughput sequencing of 18S rRNA gene. (**A**) Distribution of eukaryotic taxonomic group (dominant genera) according to DNA origin (soil or culture conditions). (**B**) Abundance of the top 5 free-living amoebae genera included in the supergroups Amoebozoa and Excavata.

As above mentioned, one key issue in FLA biodiversity assessment is the cultivability of the environmental FLA. As such, we first attempted to evaluate the diversity of FLA in Guadeloupe using culture-dependent and independent approaches (Figure 2 and Figure S1). A clear split in eukaryotic communities was revealed using principal coordinates analysis (PCoA), independently of the culture conditions (soil vs. amoeba culture at different temperatures) (permutational multivariate ANOVA methods (PERMANOVA) *p*-value < 0.001) (Figure S1A). Figure 2A presents bar plots comparing relative abundances of dominant genera without culture (DNA was extracted directly from soil) and cultivated at 30, 37 and 44 °C. In soil, ASVs were mainly assigned to Acari (76.5% of taxa), *Aspergillus* (9.9%) and *Vermamoeba* (4.2%). The use of a culture step clearly resulted in differences in both the abundance and composition of protists and other eukaryote sequences, compared to direct soil. At 30 °C, only 1.2% sequences detected corresponded to Acari, and we observed an enrichment in other genera such as *Vannella* (28.8%) *Tetrahymena* (20.1%), *Colpoda* (18%), *Rhabditida* (16.6%), and *Vermamoeba* (7.2%). At 37 °C, an increase in protist sequences is observed with predominance of *Naegleria* (24.3%), *Vermamoeba* (22.7%) and *Colpoda* (18.9%). At 44 °C, there was a substantial enrichment in *Vermamoeba* (56.1%), but also an increase in sequences corresponding to the plant *Ammopiptanthus* (23.9%), and the fungi *Aspergillus* (8%) (Figure 2A).

Figure 2B shows boxplots of abundance of specific FLA genera in the different conditions. *Vermamoeba* genus was found in all conditions, being particularly enriched at higher temperature. *Naegleria* was barely detected in soil but was found at all temperature ranges and, as expected, mainly enriched at 37 °C and 44 °C. *Vannella* was mainly detected at 30 and 37 °C while *Acanthamoeba* and *Vahlkampfia* were mainly found at 37 °C. These data were also confirmed using two complementary statistical analyses. First, the generalized linear model (GLM) showed that *Vermamoeba*, *Naegleria*, *Acanthamoeba* and *Vannella* were indeed significantly enriched when cultured at 30, 37 and 44 °C (minimum *p*-value < 0.004); this was not observed for *Vahlkampfia* (Table 1). The ANCOM (Analysis of Composition of Microbiomes) statistical analysis was then used to assess if some genera were differentially represented when comparing culture conditions. This analysis confirmed the results previously obtained with GLM: with a W value above 160, *Naegleria* (W = 183), *Vermamoeba* (W = 182), and *Vannella* (W = 174) showed significant differences in abundance levels between culture conditions. For *Acanthamoeba* (W = 149) and *Vahlkampfia* (W = 11), the differences were considered as being not significant (Figure S2). 

Afterwards, the ASV sequences corresponding to FLA were blasted (BlastN) on GenBank database (Table 2, Table S1). The genus *Vermamoeba* was represented by a single species, *V. vermiformis*, while a total of 7 putative species were characterized for *Naegleria* genus: *N. gruberi* or *N. clarki* (not differentiated by BlastN, 43.4% of *Naegleria* ASV), *N. pagei* (25.3%), *N. australiensis* (13%), *N. koreanum* (9.9%), *N. fowleri* (6.8%), *N. lovaniensis* (2.4%) and *Naegleria sp.* (2%). Concerning *Acanthamoeba* genus, an undetermined ASV corresponding to *A. castellanii* or *A. hatchetti* or *A. polyphaga* is predominant (70.3%), followed by *A. jacobsi* (10.8%), *A. byersi* (8.2%), *A. lenticulata* (7.7%) and *Acanthamoeba* sp. (3%). The ASV belonging to *Vannella* genus were distributed into *V. plantonica* (27.9%), *V. miroides* (16.9%), *V. simplex* (15.3%), *V. croatica* (14.9%), *Vannella* sp. (13%) and uncultured *Vannella* (12%). Finally, *Vahlkampfia* was mainly represented by *V. lobospinosa* (82.8%), followed by *V. avara* (15.6%) and *V. inornata* (1.6%). 

The BlastN analysis also revealed that the 18s rRNA sequences of *N. fowleri* had 100% similarity with the KY062165.1 gene sequence (Tables S1 and S2), which corresponds to a *N. fowleri* isolated from a cerebrospinal fluid (CSF) sample in China [28]. To determine *N. fowleri* genotype, the complete ITS region (ITS1, 5.8S, and ITS2) was amplified using the primers described elsewhere [10]. *N. fowleri* ITS1 sequence was found with a 100% identity with the Genbank accession number # KX909928.1, which corresponds to the genotype type 2 [29] (Table S2). 

### 2.2. Abundance and Diversity of FLA in Guadeloupe

**Figure 3:**
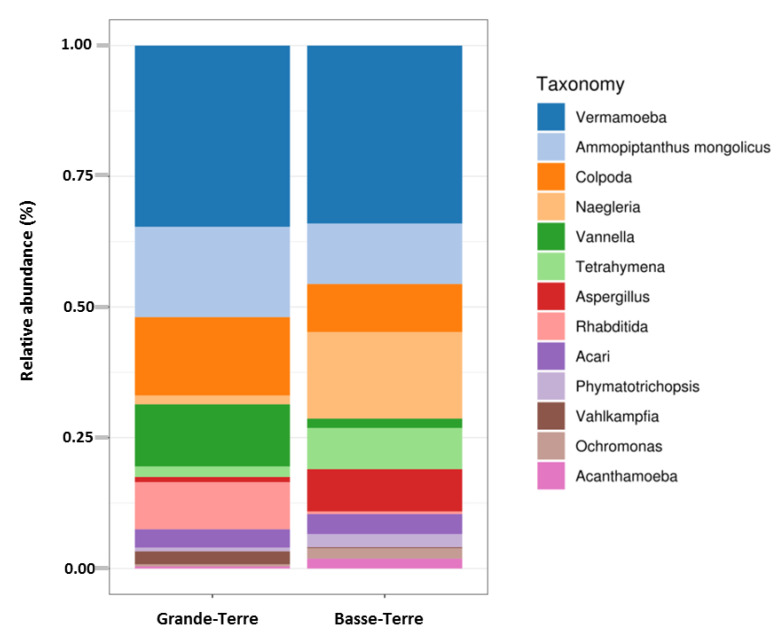
Distribution of eukaryotic taxonomic group (dominant genera) according to geographic area.

A Principal Coordinates Analysis revealed that there was a significant split between microbial communities in Basse-Terre vs. Grande-Terre islands (*p*-value = 0.001) (Figure S1B). Boxplots comparing relative abundances of FLA and dominant genera according to the two main geographical regions are presented in Figure 3. These results clearly showed that, in Basse-Terre and in Grande-Terre, the proportion of dominant genera *Vermamoeba* and *Ammopiptanthus* are relatively similar. Major differences on relative abundance are observed for *Colpoda*, *Vannella, Rhabditida* and *Vahlkampfia* (more abundant in Grande-Terre) and for *Naegleria, Tetrahymena, Aspergillus, Phymatotrichopsis, Ochromonas* and *Acanthamoeba* (more abundant in Basse-Terre).

**Figure 4:**
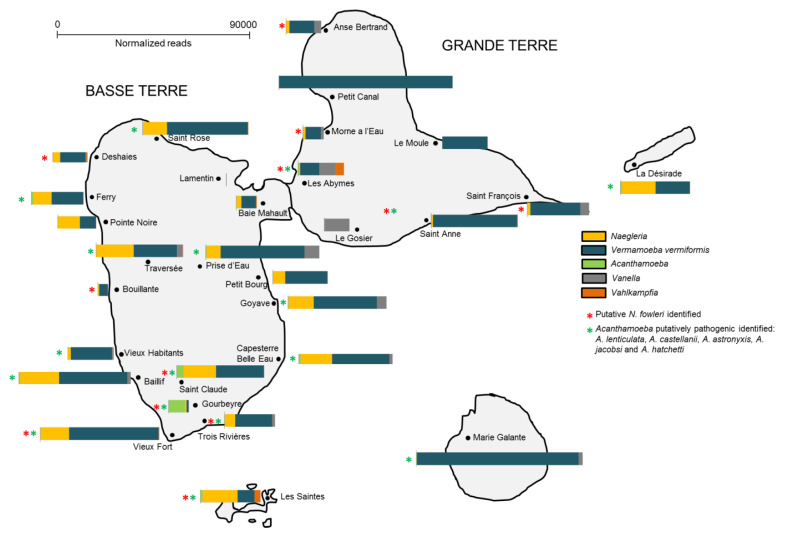
Distribution of the top 5 FLA genera according to geographic area in Guadeloupe.

Additionally, the spatial distribution of FLA in the soil within each region in Guadeloupe was shown to be variable (Figure 4). For instance, *Vermamoeba* was detected in all islands (Figure 4), being the main detected FLA in Marie-Galante, Petit-Canal and Le Moule (soil was collected near water ponds, Figure S3). High abundance of *Naegleria* sp was observed in samples collected from soil in Basse-Terre (mainly near rivers), La Désirade and Les Saintes (water ponds). Interestingly, putative *N. fowleri* species was found in 12 out of 27 sites. *Vannella* was also detected in soil collected near water ponds (Le Gosier, Anse-Bertrand, Les Abymes) and rivers (Prise d’Eau, Goyave, La Traversée). *Acanthamoeba* was mainly present in Saint Claude and Gourbeyre (near rivers) and Vahlkampfia, near two water ponds at Les Abymes and Les Saintes. *Acanthamoeba* species potentially pathogenic were detected in 17 out of 27 sites collected. 

## 3. Discussion

The diversity of FLA in the environment, and in particular in soil, is still largely unknown [1,22]. To our knowledge, this is the first study on FLA diversity and distribution in soil, using a metabarcoding approach. Indeed, most studies have focused on FLA diversity in waters [4,8,14] or on the prevalence of only one FLA genus [1].

Herein, we used a high-throughput sequencing investigation, based on the 18SrRNA gene of FLA, to provide a first overview of FLA distribution across different areas in Guadeloupe islands. For this, we firstly compared culture-dependent and independent methods; our results clearly showed that there was a significant difference in abundance and organism diversity between the two methods. This suggests that future studies on the occurrence and diversity of FLA using a similar approach should adopt culture methods. We also revealed that the spatial distribution of FLA in the soil was variable, some amoebae being prevalent in certain areas. These differences could be due to soil physical parameters such as pH, moisture (linked to the climate), and soil temperature [30]. Guadeloupe is composed of five islands. On average, the climate is tropical, hot and humid all year round. Still there are some major differences. Basse-Terre is a volcanic island with quite mountainous terrain, lush vegetation due to frequent rainfall throughout the year and with many rivers. Grande-Terre is more populated than Basse-Terre, is rather flat (resulting from the emergence of ancient coral reef) and has a drier climate, with few rivers and many ponds. The small neighboring islands Marie-Galante, La Désirade, and the small group of Iles des Saintes receive a relatively small amount of rain and are drier than Basse-Terre.

In our study, *V. vermiformis* is found to be the most abundant FLA, being widespread in Guadeloupe. This clearly shows that this amoeba is totally adapted to all types of habitats in Guadeloupe. *V. vermiformis*, together with *Acanthamoeba* and *Naegleria*, are probably the most reported free-living microorganisms around the world [1]. This amoeba has been isolated in several countries, in both anthropogenic and natural environments [25]. In addition to its ubiquity, *V. vermiformis* has gained importance due to its probable participation in pathological processes in humans and animals, through being a carrier of fungi, viruses and pathogenic bacteria such as *Bacillus anthracis*, *Legionella* and *Mycobacteria* [25,31], and its increasingly documented, apparent relationship in cases of keratitis [25,32,33].

Interestingly, our results regarding *Acanthamoeba* are unusual. As above mentioned, *Acanthamoeba* is one of the most prevalent genus of FLA found in different aquatic environments (namely in rivers and ponds [34]), being also a very abundant genus in soil (with fundamental importance in nutrient cycling) [35]. In our study, *Acanthamoeba* is scarce, being mainly detected in Saint Claude and Gourbeyre. It is widely recognized that for *Acanthamoeba* a favorable growth temperature is about 30 °C. It is thus possible that the high temperature in Guadeloupe is not favorable for their development. Interestingly, we mainly detected *Acanthamoeba* sp. that could be grown at 37 °C or even at 44 °C (such as *A. jacobsi*). The identified thermophilic *Acanthamoeba* included *A. lenticulata* [36], *A. castellanii* [37], *A. byersi* [38], *A jacobsi* [39] and *A. hatchetti* [40], all known to be involved in pathogenic processes. Unfortunately, our methodology did not allow us to identify the different *Acanthamoeba* genotypes [41].

*Naegleria* sp. were the second most abundant FLA in Guadeloupe, being mainly present in Basse-Terre. *Naegleria* sp. occur naturally in aquatic and soil habitats. More than 47 different species have been described in the genus *Naegleria*, but only one, *Naegleria fowleri*, has been associated with illness in humans [42]. In 2008, a fatal case of primary amoebic meningoencephalitis (PAM), due to *N. fowleri*, occurred in Guadeloupe, after a child swam in a bath fed with geothermal water [43]. In 2015, our group revealed that this amoeba was frequently detected in these baths [10]; the source of the organism is soil, with heavy rain causing run off into bodies of water [19]. The results presented herein show that *N. fowleri* can be found in several regions in Guadeloupe and not only in hot springs located in Basse-Terre [10]. Occurrence of *N. fowleri* in these sites can also be linked to favorable environmental parameters in the soil, such as organic matter and pH value [30,44]. According to De Jonckheere, eight different types of *N. fowleri* have been characterized based on the number of repeats in ITS1 and the C/T transition at position 31 in the 5.8S rDNA sequence [45]. In 2013, our group detected the presence *N. fowleri* with a genotype type 3 [10]. The results presented in this work revealed that the *N. fowleri* was classified as genotype 2, which has a length of 42 bp in ITS1 and the T at location 31 in the 5.8S rDNA. As such, the two genotypes coexist in Guadeloupe islands. Besides *N. fowleri*, *N. australiensis* and *N. italica* can cause PAM in experimental infections in animals (cows, donkeys, fish, greyhounds, mice, etc.) [46]. Herein, we were not able to detect any *N. italica*, but we found *N. australiensis*.

The third most detected FLA in Guadeloupe was the *Vannella* genus. Although it was found in several parts of the islands, it was mainly detected at Le Gosier and Les Abymes. Members of the genus *Vannella* occur in aquatic environments, soils, and as the epi- and endobionts of vertebrate hosts [47,48,49,50]. Although *Vannella* spp. is not considered so far as a pathogen for humans, like other FLA it can facilitate the growth of bacteria [51,52] including *Legionella* [53], *Pseudomonas aeruginosa* [54], and other organisms [55], some of which are human pathogens. The genus has also been implicated with taste and odor problems in drinking water by the production of geosmin [56]. 

*Vahlkampfia* genera is one of the most frequently found in the environment [5]. Herein, we were able to detect this FLA mainly in one water pond in Les Abymes, and with a low relative abundance. As for *Acanthamoeba*, it is possible that the soil conditions are not favorable for the development and/or maintenance of this genus.

Although our primers allowed the identification of a wide range of eukaryotic organism in our samples, it is possible that we have missed some non-cultivable FLA. The use of PCR (instead of direct sequencing) can also bring some drawbacks (due to the choice of primers) to accurately describing FLA diversity and abundance; this is mostly due to the variations of 18SrRNA copy number found in various FLA [2]. Another important parameter to consider is the variations in DNA yield, depending on the amoeba growth stage [57,58]. Although some FLA trophozoites have been found in the soil [1], cysts are considered the predominant stages in this habitat, which could result in a lower yield of DNA. Conversely, the culture method allows an increase in the number of FLA stages, besides being favorable to trophozoites. To avoid this bias in our analyses, we collected our samples (after the culture enrichment steps) when the amoeba were in the cyst form. 

## 4. Materials and Methods

### 4.1. Study Sites and Soil Sampling

In this study, 107 soil samples were collected from the five islands of Guadeloupe: Basse-Terre (22 sites, 62 samples), Grande-Terre (15 sites, 39 samples), La Désirade, Les Saintes and Marie-Galante. Soil was sampled mainly near rivers and hot springs (in Basse Terre) and natural ponds (in Grande Terre, La Désirade, Les Saintes and Marie-Galante) in January-February (dry season) 2018 and October (rainy season) 2018 (Figure S3 and Table S3). The surface layer (0–10 cm) was collected, after removing plants, pebbles and conspicuous roots. At each site, three samples were taken at least 3 m apart. Samples were kept at room temperature and processed within 24 h, for DNA extraction or amoeba culture. 

### 4.2. Amoebal Culture

For each replicate per site, four spots of fresh soil (100 µg/spot) were placed onto NNA (Non-Nutrient Agar) plates seeded with live *Escherichia coli*, as described previously [19]. The plates were incubated at 30, 37 and 44 °C to allow different species of cultivable FLA to grow. The plates were monitored daily with an inverted phase contrast microscope for amoebic growth. Samples were considered negative if no amoebal development could be observed by microscopy after two weeks. The presence of amoebal cells was characterized by the formation of a migration front, representing the amoebal development upon agar plates. Samples were recovered by scrapping the agar plate with 800 µL of T1 Lysis Buffer (from the kit NucleoSpin Tissue, Macherey Nagel, Germany) and a cell scraper for DNA extraction post-cultivation. “T1-cell suspension” was kept at −20 °C until further use.

### 4.3. DNA Extraction

#### 4.3.1. DNA Extraction Directly from Soil

DNA extraction from the soil samples was carried out using the NucleoSpin^®^ Soil DNA extraction kit (Macherey-Nagel, Duren, Germany), employing SL1 lysis buffer and extraction enhancer SX, according to manufacturer’s recommendations. DNA was stored at −20 °C until further processing.

#### 4.3.2. DNA Extraction from FLA Cultures

T1-cell suspensions (180 μL) were treated with proteinase K (25 µL) for 2 h at 56 °C. Afterwards, the samples went through a DNA extraction protocol using the NucleoSpin^®^ Tissue DNA extraction kit (Macherey-Nagel, Germany), following the manufacturer’s recommendations. DNA was stored at −20 °C until use.

### 4.4. Amplicon Libraries Preparation and Sequencing

DNA samples were amplified using in-house developed 18S primers MGA-F: 5′- TGCGGCTTAATTYGACTCAAC-3′ and MGA-R: 5′-GCATCACAGAYCTGTT-3′ targeting a 273-443 bp fragment, depending on the FLA species (Table S4). Primers were synthesized with nucleotidic adaptors for Illumina sequencing. Amplification was carried out with a PCR mix (10× buffer, Eurobio) containing 3 mM MgCl_2_ and 0.2 mM of dNTP, 0.4 µM of each primer, 5% DMSO, 0.4 mg/ml of BSA, 0.05 U/µL of Taq DNA polymerase, and 50–100 ng of genomic DNA in a final volume of 50 µL. Amplification conditions were: 5 min pre-heating at 95 °C, followed by 40 cycles at 94 °C for 45 s, 50 °C for 1 min, 72 °C for 2 min, and final extension for 10 min at 72 °C. PCR analysis was performed using a 2720 Thermal Cycler (Applied Biosystems). 

Aliquots (10 µL) of each PCR products were mixed with 2 µL of loading buffer (10 mM EDTA, 10% glycerol, 0.015% bromophenol blue, 0.17% SDS), run in a 1.5 % agarose gel in TBE buffer (89 mM Tris, 89 mM Boric Acid, 2 mM EDTA, pH 8.3) and visualized with GelRed^®^ Nucleic Acid Gel Stain (Biotium). A 100-bp DNA ladder (Invitrogen) was used as a size marker in the gels. Negative DNA controls (template DNA replaced with distilled water), and positive controls (*Acanthamoeba jacobsi*, *Vermamoeba vermiformis*, *Naegleria fowleri*, *N. lovanienesis*, *N. australiensis*, *Willaertia magna*) were used. Positive amplicons were pooled according to their sampling site and in-gel purified using QIAQuick gel extraction (Qiagen, Germany). 

Purified amplicons were then checked and sized using a bioanalyser coupled with DNA 1000 kit (Agilent Technologies). DNA concentration was assessed using Quant-It PicoGreen dsDNA kit (Life Technologies), and each amplicon was adjusted at a concentration of 10^9^ DNA molecules/μL before pooling. The following steps were conducted as recommended by the Amplicon Library Preparation manual provided by Roche, and samples were loaded on a GS junior pyrosequencer (Roche).

Paired-end sequencing (2 × 300 bp) of the 18S rRNA was performed with the Illumina MiSeq System. The read sequences from this study were deposited to Genbank (Accession number PRJNA624062). 

### 4.5. Bioinformatics Analyses-Metabarcoding

Initial reads quality inspection was performed with FastQC (https://www.bioinformatics.babraham.ac.uk/projects/fastqc). Paired-end reads were trimmed and filtered using AlienTrimmer [59] with a quality Phred score threshold of 28 on a minimum length of 70 nucleotides. Denoising and chimera removal was performed using DADA2 software package [60] implemented in QIIME 2 [61] *via* q2-dada2 plugin. DADA2 allows fine-scale variation identification through the characterization of amplicon sequence variants (ASV). Details on DADA2 filtering statistics are available in Supplementary Materials. Singletons and rare ASV (bellow 0.001%) were removed thanks to q2-feature-table plugin leading to 817 features for a total of 9211614 reads, a median frequency of 63490 reads per samples and a mean read length of 240 bp (± SD 46). Taxonomy was assigned to ASV using the q2-feature-classifier [62], a classify-sklearn naive Bayes taxonomy classifier using machine learning [63] against the Silva 132 99_18S database [64]. A heatmap was performed on collapsed feature table to taxonomic level 6 (corresponding genus) using Matplotlib [65] via q2-feature-table. Normalization of ASV table was done by DESeq2 [66] implemented in the SHAMAN pipeline [67] as suggested by McMurdie and Holmes [68] (Table S5). Alpha diversities were calculated using the Shannon index. Beta-diversity was calculated from the DESeq2 normalized data by a Bray-Curtis dissimilarity measure. Community structure differences among samples were visualized using principal coordinates analysis (PCoA) against two variables: temperature of culture (no culture, 30 °C, 37 °C and 44 °C) and region of sampling in Guadeloupe (“Basse Terre” vs. “Grande Terre”). Effects of these variables on beta diversity were tested with permutational multivariate ANOVA methods (PERMANOVA) with 999 permutations of the Bray-Curtis distance matrix. The generalized linear model (GLM) was then applied to detect differences in abundance of genera between variables tested with Benjamini-Hochberg correction. An ANCOM (Analysis of Composition of Microbiomes) [69] was performed using q2-composition plug-in on ASV table collapsed to genus taxonomic level and comparing culture conditions: it is a compositional approach that makes no assumptions about feature distributions. Finally, ASV sequences corresponding to FLA were blasted on GenBank public database [70] using the BlastN algorithm [71] in order to potentially identified amoebas at the species level. 

Workflow used for this metabarcoding is presented as (Supplemental Figure S4).

## 5. Conclusions

This work presents the diversity, distribution and relative abundance of FLA present in soil in Guadeloupe Islands, using a culture-based approach and metagenomics (using in-house developed new primers targeting the 18S regions of FLA). Our results highlighted dominant genera such as *Vermamoeba* and *Naegleria*. Although most FLA identified in this study were non-pathogenic (*Vermamoeba*, *Vannella*, *Vahlkampfia* and *Naegleria*, besides *N. fowleri*), their presence in soil represents a potential hazard, since some species of these genera can act as reservoirs for the presence and transmission of other human pathogens [2]. Further investigations should be conducted to better understand their ecology and their possible impact on human and animal health. 

## Figures and Tables

**Table 1:** Differences in normalized Amplicon Sequence Variant (ASV) abundances revealed by generalized linear model (GLM) and false discovery rate (FDR) according to the culture-dependent and independent conditions and the geographical area.

Variable	Genus	Base Mean	Fold Change	Log2 Fold Change	*p*-Value Adjusted
T 44 °C vs. soil (no culture)	*Vermamoeba*	17,284.4	60.1	5.9	1.4 × 10^−14^
	*Naegleria*	5773.1	245.3	7.9	6.4 × 10^−12^
	*Acanthamoeba*	644.4	96.2	6.6	4.7 × 10^−3^
	*Vannella*	2385.1	86.1	6.4	1.1 × 10^−3^
T 37 °C vs. soil (no culture)	*Vermamoeba*	17,284.4	15.4	3.9	4.4 × 10^−7^
	*Naegleria*	5773.1	655.3	9.4	5.4 × 10^−17^
	*Acanthamoeba*	644.4	1722.2	10.8	4.8 × 10^−7^
	*Vannella*	2385.1	1790.0	10.8	2.25 × 10^−9^
T 30 °C vs. soil (no culture)	*Vermamoeba*	17,284.4	4.3	2.1	4.7 × 10^−2^
	*Naegleria*	5773.1	54.1	5.8	6.9 × 10^−5^
	*Acanthamoeba*	644.4	280.7	8.1	2.0 × 10^−3^
	*Vannella*	2385.1	6855.7	12.7	5.9 × 10^−9^
Basse Terre vs. Grande Terre	*Naegleria*	5773.1	6.2	2.6	3.7 × 10^−13^

**Table 2:** BlastN characterization of ASV corresponding to free-living amoebae (FLA) genera *Vermamoeba*, *Naegleria*, *Acanthamoeba*, *Vanella* and *Vahlkampfia* against GenBank database. (Details on BlastN results are presented in Table S1).

Genus	BlastN Species	Reads Count	% in Genus
*Vermamoeba*	*V. vermiformis*	1,849,430	100
*Naegleria*	*Naegleria gruberi/Naegleria clarki*	264,610	43.4
	*Naegleria pagei*	154,394	25.3
	*Naegleria australiensis*	78,993	13
	*Naegleria koreanum*	60,074	9.9
	*Naegleria fowleri*	41,286	6.8
	*Naegleria lovaniensis*	14,617	2.4
	*Naegleria* sp.	11,959	2
*Acanthamoeba*	*Acanthamoeba castellanii/Acanthamoeba hatchetti/Acanthamoeba polyphaga*	48,447	70.3
	*Acanthamoeba jacobsi*	7451	10.8
	*Acanthamoeba byersi*	5665	8.2
	*Acanthamoeba lenticulata*	5311	7.7
	*Acanthamoeba* sp.	2080	3
*Vannella*	*Vannella planctonica*	71,125	27.9
	*Vannella miroides*	43,178	16.9
	*Vannella simplex*	39,033	15.3
	*Vannella croatica*	38,115	14.9
	*Vannella* sp.	33,155	13
	*Uncultured Vannella*	30,604	12
*Vahlkampfia*	*Vahlkampfia lobospinosa*	43,653	82.8
	*Vahlkampfia avara*	8211	15.6
	*Vahlkampfia inornata*	847	1.6

## Supplementary Materials

The following are available online at https://www.mdpi.com/2076-0817/9/6/440/s1, Figure S1: PCoA based on Bray-Curtis dissimilarity matrix of normalized ASV data revealing community structures differences (validated by permanova test *p*-value < 0.001) against (A) cultures conditions at 30, 37 and 44 °C and (B) sampling region in Basse-Terre vs. Grande-Terre islands., Figure S2: FLA genera significantly contrasted between culture conditions according to ANCOM (Analysis of Composition of Microbiomes). W reflect the effect size difference of a given genus between culture conditions, and clr the F-score traduce the strength of the ANCOM test statistic. The ANCOM analysis revealed that Vermamoeba, Naegleria, Acanthamoeba and Vannella present differences in abundance depending on the culture conditions, Figure S3: Map of Guadeloupe with the location of some of the soil sampling areas and showing the different ecosystems, Figure S4: Workflow for FLA metabarcoding analyses, Table S1: ASV BLASTN assignments of FLA against NCBI database. Only first three BlastN results are presented when several results are possible for the same nucleotidic identity and query coverage, Table S2: 18S rRNA and ITS1 sequences of Naegleria fowleri isolates identified in soil samples Guadeloupe, Table S3: Localization and Global Positioning System (GPS) coordinates of the soil samples used in this study, Table S4: Predicted FLA amplicons size using CLC Seq Viewer and primers MGA R2 and F2, Table S5: Normalization of ASV genus level performed with DESeq2.
