# Supplementary material for: Cartography of Free-Living Amoebae in Soil in Guadeloupe (French West Indies) Using DNA Metabarcoding
Source: Pathogens. 2020 Jun 4;9(6):440. doi: 10.3390/pathogens9060440 (PMC7350318; doi:10.3390/pathogens9060440)
Supplement: Supplementary file 1 [file pathogens-09-00440-s001.zip › Reynaud et al_suppl/Table S4_amplicon size.docx]

**Supplemental Table 4**: predicted FLA amplicons size using CLC Seq Viewer and primers MGA R2 and F2

| FLA species | Identifiant GenBank | Size of gene rRNA 18S (bp) | Localization of *forward* primer on target gene | Localization of *reverse* primer on target gene | Average size of the amplicons (bp) |
| --- | --- | --- | --- | --- | --- |
| *Naegleria fowleri* | AF338423.1 | 1988 | 1331 | 1647 | 316 |
| *Naegleria lovaniensis* | U80062.1 | 1981 |  |  |  |
| *Acanthamoeba castellanii* | AF251938.1 | 2290 | 1492 | 1854 | 362 |
| *Acanthamoeba rhysodes* | U07406.1 | 2280 |  |  |  |
| *Acanthamoeba polyphaga* | U07407.1 | 2287 |  |  |  |
| *Balamuthia mandrillaris* | AF019071.1 | 1973 | 1272 | 1616 | 344 |
| *Tetramitus thermacidophilus* | KM669765.1 | 1941 | 795 | 1121 | 326 |
| *Hartmanella abertawensis* | DQ190241.1 | 1840 | 1194 | 1467 | 273 |
| *Sappinia platani* | EU881943.1 | 2507 | 1624 | 2067 | 443 |
