# Supplementary figures and images for: Cartography of Free-Living Amoebae in Soil in Guadeloupe (French West Indies) Using DNA Metabarcoding
Source: Pathogens. 2020 Jun 4;9(6):440. doi: 10.3390/pathogens9060440 (PMC7350318; doi:10.3390/pathogens9060440)

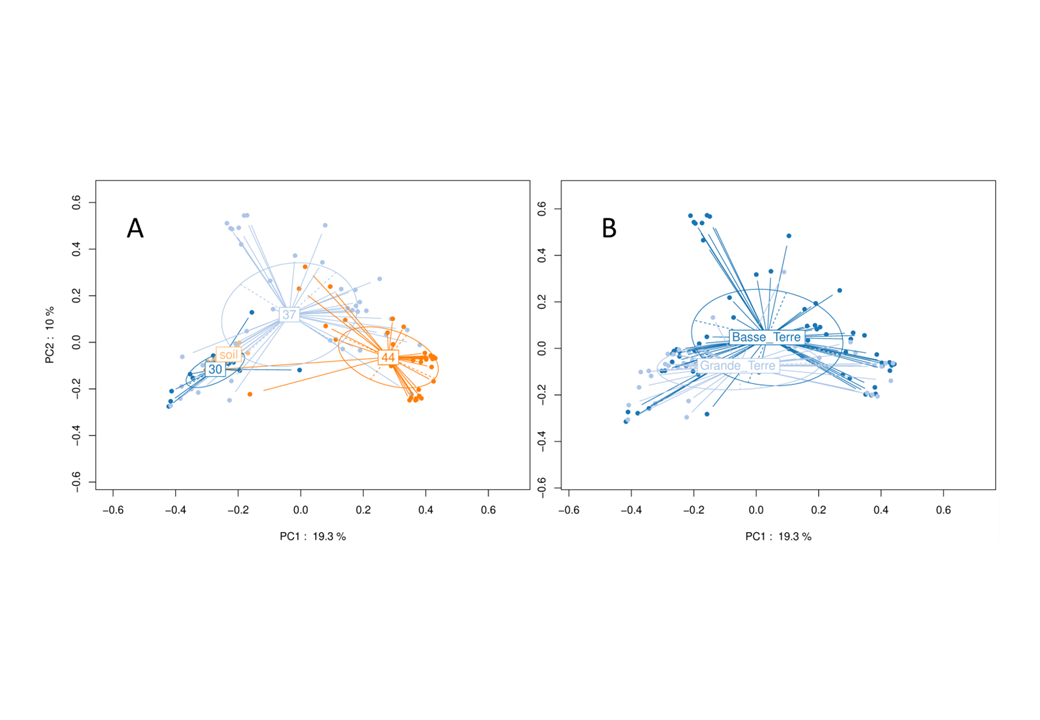

Supplement: Supplementary file 1 [file pathogens-09-00440-s001.zip › Reynaud et al_suppl/Figure S1_PCoA.png]

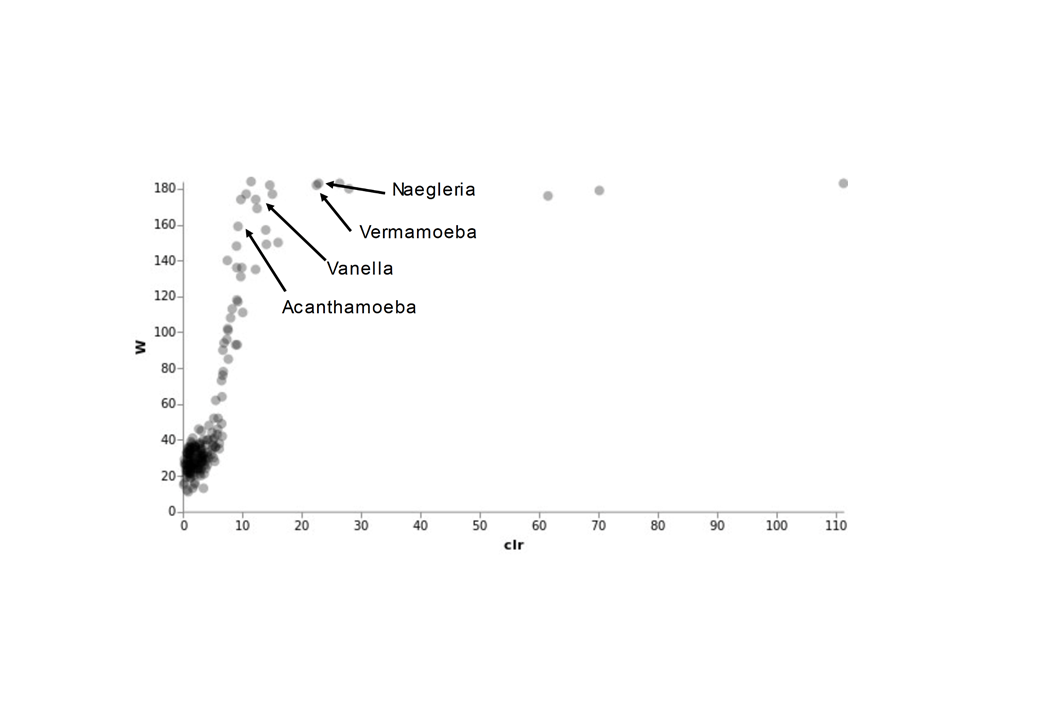

Supplement: Supplementary file 1 [file pathogens-09-00440-s001.zip › Reynaud et al_suppl/Figure S2_ANCOM.png]

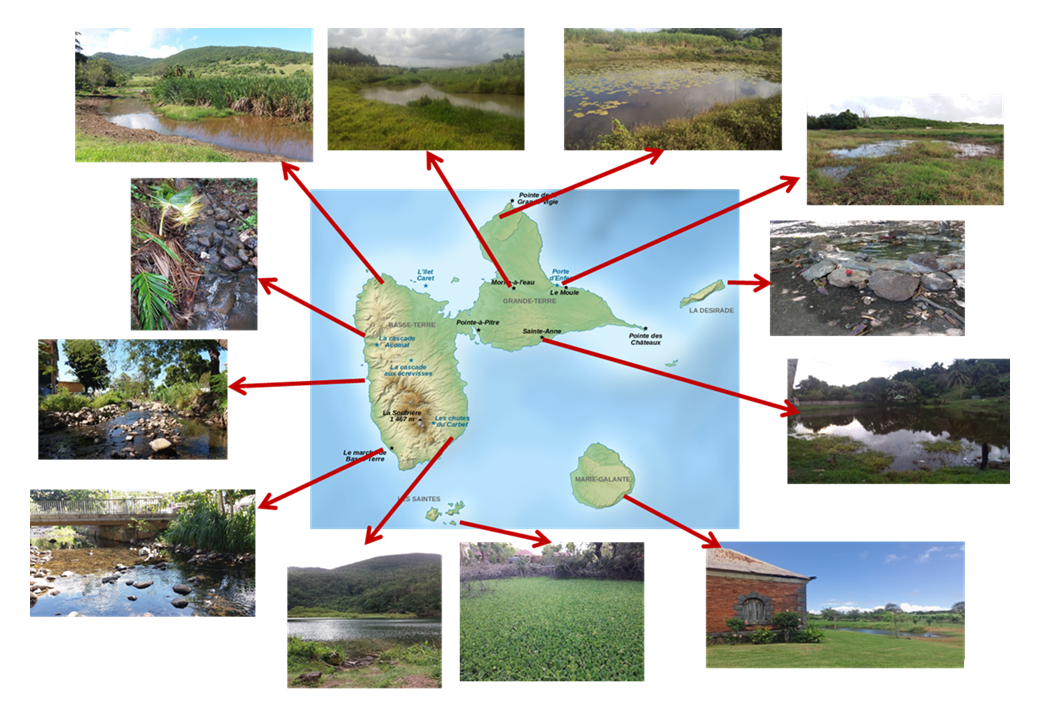

Supplement: Supplementary file 1 [file pathogens-09-00440-s001.zip › Reynaud et al_suppl/Figure S3_ecosystems_soil.png]

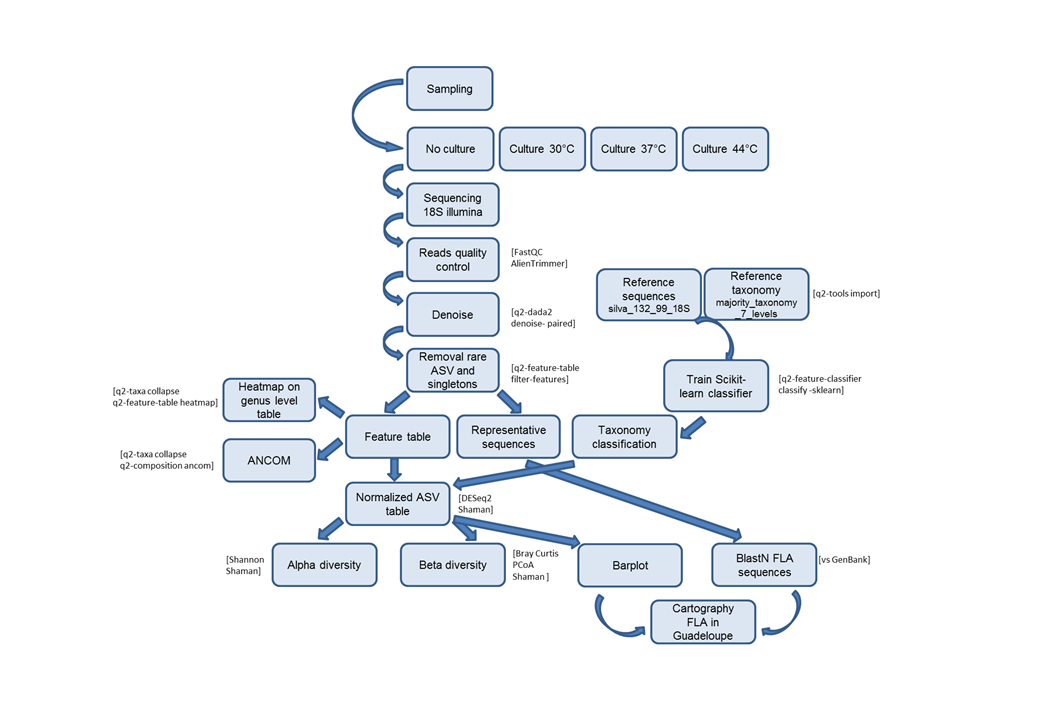

Supplement: Supplementary file 1 [file pathogens-09-00440-s001.zip › Reynaud et al_suppl/Figure S4_workflow.png]
